# Dental, skeletal and soft tissue changes after bimaxillary protrusion treatment with temporary anchorage devices using different retraction mechanics

**DOI:** 10.1186/s12903-024-03927-1

**Published:** 2024-01-27

**Authors:** Kotoe Mayahara, Satoru Kawai, Tomihiro Fujisaki, Noriyoshi Shimizu

**Affiliations:** 1 Department of Orthodontics, Nihon University School of Dentistry, 1-8-13, Kanda Surugadai, Chiyoda-ku, Tokyo, 101-8310 Japan; 2 Division of Clinical Research, Dental Research Center, Nihon University School of Dentistry, Tokyo, Japan; 3 Private practice, Fukuoka, Japan; 4 Private practice, Tokushima, Japan

**Keywords:** Temporary anchorage device, Bimaxillary protrusion, Closing loop

## Abstract

**Background:**

Temporary anchorage devices (TADs), which are absolute anchorage, are used for retraction of the anterior teeth in cases of severe bimaxillary protrusion. There have been a number of studies regarding anterior tooth movement using TADs performed by simulation systems and actual treated materials with sliding mechanics. However, there are few studies regarding anterior tooth movement using TADs treated by loop mechanics The purpose of this study was to investigate the effect of TADs in anterior tooth movement using loop mechanics performed in actual cases of bimaxillary protrusion.

**Methods:**

This study was performed in 20 adult patients with severe bimaxillary protrusion treated with four bicuspid extraction with sliding or loop mechanics (*n* = 10 in each mechanics) using TADs. The skeletal and denture patterns, as well as the soft tissue profile from pre-treatment (T0) and post-treatment (T1) lateral cephalograms, were compared between sliding and closing loop mechanics.

**Results:**

The use of TADs is useful for retraction of anterior teeth without molar anchorage loss. in sliding and loop mechanics. The upper anterior teeth were less lingual tipped and lower anterior teeth were more upright resulting in less clockwise rotation of the occlusal plane in loop mechanics compared to sliding mechanics.

**Conclusion:**

An oblique retraction force vector with a lower point of application causes less intrusion and more lingual tipping of upper anterior teeth as well as more clockwise rotation of the occlusal plane compared to a parallel retraction force vector.

## Introduction

Patients with severe bimaxillary protrusion are among the most difficult cases in orthodontic treatment because not only occlusion but also the lateral profile must be improved. For successful treatment in cases of severe bimaxillary protrusion, it is essential to use all the extraction space of the bicuspids for anterior tooth retraction without molar mesial movements. A J-hook headgear has been used as an anchorage device in such severe cases to achieve sufficient retraction as well as intrusion of the maxillary anterior teeth; This leads to a counterclockwise rotation of the occlusal plane and contributes the improvement of a gummy smile [1, 2]. However, patient cooperation is essential to obtain a better treatment result and insufficient patient compliance with using the headgear leads to poor result [3, 4].

In the last decade, there has been significant development of Temporary Anchorage Devices (TADs) for achieving absolute anchorage, and they have been used particularly in severe cases [5–7]. The use of TAD facilitates the treatment of bimaxillary protrusion, causes good results. TADs have recently been implanted in various sites [8–10], but we often perform implantation into the buccal alveolar bone of the molar area in cases of bimaxillary protrusion to retract anterior teeth. Anterior tooth retraction in cases of bimaxillary protrusion is usually accomplished with sliding or closing loop mechanics [11, 12]. Straight arch wire is used in sliding mechanics for its clinical simplicity, and it is usually smaller size to prevent friction and binding. Conversely, it does not provide good torque control of the anterior teeth [11]. Loop mechanics has the advantage of facilitating torque control of the anterior teeth because a larger arch wire can be used without friction and binding [11].

In the treatment of bimaxillary protrusion using TADs, it is necessary to examine the behavior of the anterior tooth movement according to the tow direction from TADs in each mechanics. Although there have been reports regarding anterior tooth movement according to the tow direction from TADs implanted in the buccal molar area, many studies were not used actual data from patients but performed by simulation using finite element modeling of sliding mechanics [13–16], also there have been few studies of loop mechanics. Only a few case reports [17, 18] treated loop mechanics are shown, and there are no studies discussed the behavior of the anterior tooth movement with loop mechanics using TADs in actual data through orthodontic patients. In this study, to clarify the effect of different tow direction from TADs in anterior tooth retraction, we examined the behavior of anterior tooth movement of 20 patients with severe bimaxillary protrusion treated by sliding or loop mechanics.

## Materials and methods

**Fig. 1:**
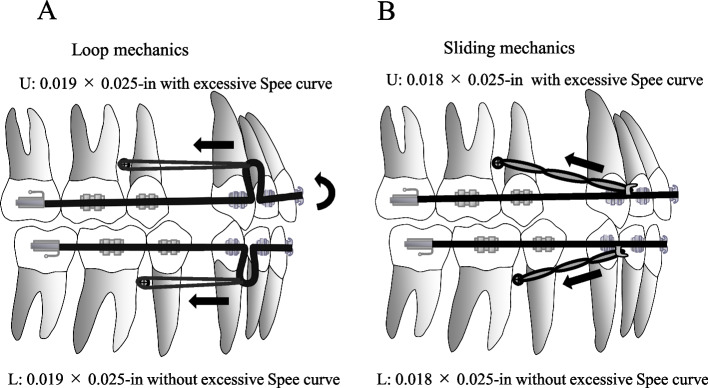
Schematic diagram of the sliding and loop mechanics. Schematic diagram of the loop and sliding mechanics. **A** the anterior teeth were retracted with a closing loop bent between the lateral and canine on a 0.019 × 0.025-in stainless steel arch wire with excessive Spee curve only for the maxillary arch. **B** sliding mechanics, the anterior teeth were retracted with a power chain from a hook fixed between the lateral and canine on a 0.018 × 0.025-in stainless steel arch wire with excessive Spee curve only for the maxillary arch

This study was performed with the approval of the Ethics Committee of Nihon University School of Dentistry (EP16D021) and all the methods included in this study are in accordance with the declaration of Helsinki. The study population consisted of 20 adult patients aged 16–35 years (3 male and 17 female) with severe bimaxillary protrusion with an Angle’s Class 1 M relationship, no more than 3.0 mm of anterior crowding, and 15–16 mm of total discrepancy. They had no previous history of orthodontic treatment, no periodontal disease, and no missing teeth. All were treated with extraction of the four first bicuspids and placement of the four TADs in the buccal alveolar bone between the second bicuspid and first molar to provide anchorage. TADs 1.6 mm in diameter and 8 mm in length (ISA; Biodent, Tokyo, Japan) were implanted at a height of 5–6 mm above the gingival margin just under the contact point between the second bicuspid and the first molar using the self-drilling method or the self-tapping method with a drilled pilot hole (diameter, 1.0 mm) before placing the TADs. The TADs in all subjects included were stable without mobility during the treatment period. They were treated with a full fixed 0.022-in slot standard edgewise bracket (Tomy International Inc., Tokyo, Japan) in four steps: alignment and leveling of the mid-arch, canine retraction for secure anterior tooth space and re-leveling of all teeth, full anterior tooth retraction, and occlusion finishing and detailing. The subjects were randomly divided into two groups (*n* = 10 in each group) depending on the treatment mechanics. Patients in the loop group were treated with closing loop mechanics in which the anterior teeth were retracted with a closing loop (size: 8.0 mm × 3.5 mm) bent between the lateral and canine on a 0.019 × 0.025-in stainless steel archwire with excessive Spee curve only for the maxillary arch (Fig. 1A). In the sliding group treated with sliding mechanics, the anterior teeth were retracted with a power chain (4 oz. each side) from a hook of 3 mm in height fixed between the lateral and canine on a 0.018 × 0.025-in stainless steel archwire with excessive Spee curve only for the maxillary arch (Fig. 1B). The average ages of the patients in the two groups were 21 years 9 months and 22 years 5 month, respectively.

**Fig. 2:**
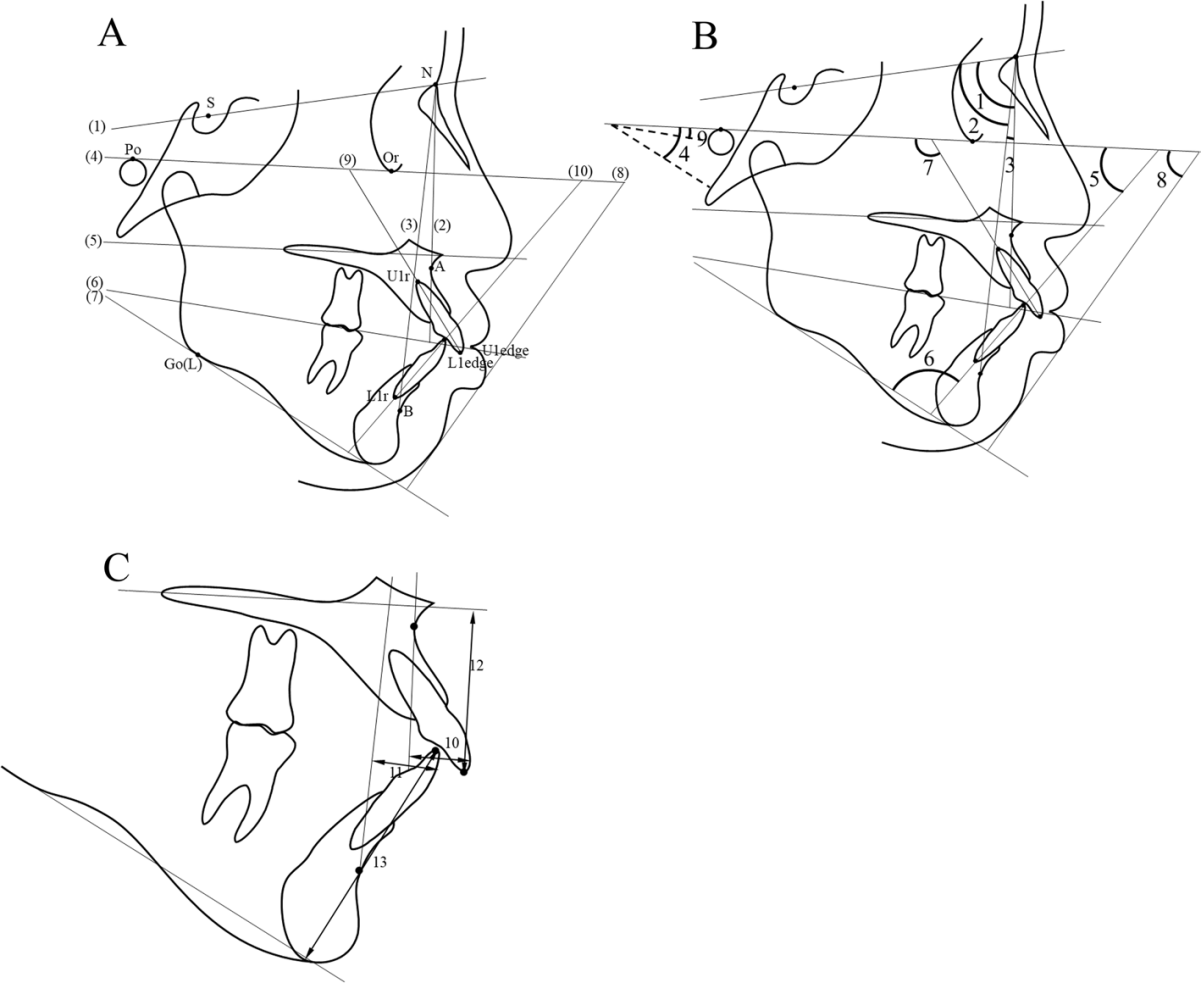
Cephalometric measurements. **A** Landmarks and reference planes: Sella (S), Nasion (N), A point (A), B point (B), surface of upper central incisor (U1s), surface of lower central incisor (L1s), edge of upper central incisor (U1edge), edge of lower central incisor (L1edge), root apex of upper central incisor (U1r), root apex of lower central incisor (L1r), gonion on mandibular plane (Go(L)), menton (Me), (1) SN plane; (2) NA plane; (3) NB plane; (4) FH plane; (5) palatal plane; (6) occlusal plane; (7) Go(L)-Meplane; (8) profile line; (9) line of U1edge-U1r; (10) line of L1edge-L1r. **B** Angular measurements; 1, SNA; 2, SNB; 3,ANB; 4,FMA; 5, FMIA; 6, IMPA; 7,FH to U1; 8, Z-angle; 9, FH to Occ.P. **C** Linear measurements; 10, NA to U1; 11, NB to L1; 12, U1 edge to PP; 13, L1 edge to MP

Pre-treatment (T0) and post-treatment (T1) lateral cephalograms were obtained for all subjects. Tracing, superimposition, and measurement were performed manually by two examiners. The cephalometric parameters, including five skeletal and seven denture measurements, and one soft tissue profile were used in this study (Fig. 2). Standard cephalometric landmarks and reference planes are shown in Fig. 2A. The cephalometric measurements used in this study are shown in Table 1 and Fig. 2B, C. The variation from T1 to T0 (T1–T0) was calculated for each subject.

**Table 1:** Definitions of cephalometric measurements

Measurement	Definition
SNA (°)	Angle between SN plane and NA plane
SNB (°)	Angle between SN plane and NB plane
ANB (°)	Difference between SNA and SNB
FMA (°)	Angle between FP plane and Go(L)-Me plane
FMIA (°)	Angle between FH plane and line of L1edge-L1r
IMPA (°)	Angle between mandibular plane and line of L1edge-L1r
FH to U1 (°)	Angle between FH plane and line of Uedge-U1r
NA to U1 (mm)	Linear distance from NA plane to U1s
NB to L1 (mm)	Linear distance from NB plane to L1s
Z-angle (°)	Angle between FH plane and profile line
U1 edge to PP (mm)	Linear distance from U1edge to palatal plane
L1 edge to MP (mm)	Linear distance from L1edge to mandibular plane
FH to Occ. P (°)	Angle of FH plane and occlusal plane

### Statistical analysis

To evaluate intraexaminer and interexaminer reliabilities, the cephalograms were retraced and remeasured by the same two examiners after 3-month interval. The analysis results revealed no statistically significant differences between the first and second measurements at the 0.05 significance level. All data are shown as means ± standard deviation, and differences between the two groups were validated by Student’s *t-*test. In all analyses, *P* < 0.05 was taken to indicate statistical significance. All statistical tests were performed using SPSS software version 16.0 (SPSS Japan, Tokyo, Japan).

## Results

There were no significance differences in any of the mean measurements between the two groups at T0. Treatment results for the two methods were compared with regard to horizontal and vertical cephalometric measurements.

### Horizontal cephalometric measurements (Table 2)

In both groups, the dental and soft tissue measurements were significantly different between T0 and T1 (T0 vs. T1), while skeletal measurements showed no significant difference.

**Table 2:** Horizontal cephalometric measurements

Variable	T0			T1			Significance of T0 vs. T1		T1ーT0		
	Loop Mean SD	Sliding Mean SD	Sig.	Loop Mean SD	Sliding Mean SD	Sig.	Loop	Sliding	Loop Mean SD	Sliding Mean SD	Sig.
SNA (°)	81.8 (3.9)	82.1 (2.9)	NS	81.7 (3.9)	82.0 (3.0)	NS	NS	NS	−0.2 (0.7)	−0.1 (0.3)	NS
SNB (°)	78.2 (4.6)	77.4 (3.2)	NS	77.6 (4.9)	76.8 (3.1)	NS	NS	NS	−0.6 (0.6)	−0.7 (0.5)	NS
ANB (°)	3.6 (1.5)	4.7 (1.3)	NS	4.0 (1.8)	5.3 (1.4)	NS	NS	NS	0.4 (0.7)	0.6 (0.6)	NS
FMA (°)	27.9 (6.4)	29.6 (5.1)	NS	27.9 (6.5)	29.9 (5.0)	NS	NS	NS	0.0 (1.3)	0.3 (1.1)	NS
FM IA (°)	50.0 (6.9)	48.0 (4.9)	NS	67.5 (8.2)	59.5 (3.9)	*	**	**	17.5 (7.1)	11.5 (4.7)	*
IMPA (°)	102.2 (8.4)	102.7 (7.6)	NS	84.7 (8.8)	90.7 (5.7)	NS	**	**	−17.5 (7.1)	−12.0 (4.8)	NS
FH to U1 (°)	121.8 (5.8)	124.1 (3.3)	NS	106.8 (6.5)	103.0 (3.4)	NS	**	**	−15.0 (6.4)	−21.1 (4.7)	*
NA to U1 (mm)	9.0 (2.4)	10.6 (2.1)	NS	1.2 (2.3)	1.4 (2.9)	NS	**	**	−7.8 (1.2)	−9.2 (1.7)	NS (*P* = 0.054)
NB to L1 (mm)	10.8 (1.7)	11.5 (2.0)	NS	3.6 (1.0)	5.8 (1.3)	**	**	**	−7.1 (1.2)	−5.8 (1.3)	*
Z-angle (°)	63.5 (6.7)	59.3 (3.1)	NS	77.2 (5.1)	70.7 (5.0)	*	**	**	13.8 (3.5)	11.4 (4.2)	NS

^*^p<0.05, ^**^p<0.01

Comparing the values of T1 between two groups, significant differences were found in the FMIA, NB to L1, and Z-angle between the two groups.

Comparing the variation of T1-T0 (absolute value of tooth movement) between loop and sliding groups, the FMIA of the loop group was significantly larger (17.5°) than that of the sliding group (11.5°). NB to L1 in the loop group was significantly larger (7.1 mm) than that in the sliding group (5.8 mm). These results indicate that the lower incisor was significantly uprighted by loop mechanics compared to sliding mechanics. But FH to U1 in the sliding group was significantly larger (21.1°) than that in the loop group (15.0°), indicating more lingual crown tipping of the upper incisor in the sliding group.

### Vertical cephalometric measurements (Table 3)

Comparing the values of T0 and T1 (T0 vs. T1), L1 edge to MP was significantly decreased in the sliding group, and at T1, significant difference was found in the FH to Occ. P between two groups. Comparing the variation of T1-T0 between loop and sliding groups, U1 edge to PP and FH to Occ. P were significantly smaller in the loop group (− 1.3 mm; 0.3°) than in the sliding group (0.7 mm; 3.5°). These results indicate that the upper incisor exhibited more intruded and FH to Occ. P exhibited less clockwise rotation in the loop group than the sliding group.

**Table 3:** Vertical cephalometric measurements

Variable	T0			T1			Significance of T0 vs. T1		T1–T0		
	Loop Mean SD	Sliding Mean SD	Sig.	Loop Mean SD	Sliding Mean SD	Sig.	Loop	Sliding	Loop Mean SD	Sliding Mean SD	Sig.
U1 edge to PP (mm)	33.1 (2.3)	31.3 (1.9)	NS	31.8 (3.1)	32.0 (1.6)	NS	NS	NS	−1.3 (1.8)	0.7 (1.5)	*
L1 edge to MP (mm)	48.6 (3.3)	48.0 (3.8)	NS	45.9 (3.2)	43.9 (4.2)	NS	NS	*	−2.7 (2.3)	−4.1 (1.5)	NS
FH to Occ. P (°)	7.2 (4.0)	8.5 (2.5)	NS	7.6 (3.9)	12.0 (4.6)	*	NS	NS(*p* = 0.053)	0.3 (2.3)	3.5 (2.6)	**

**Fig. 3:**
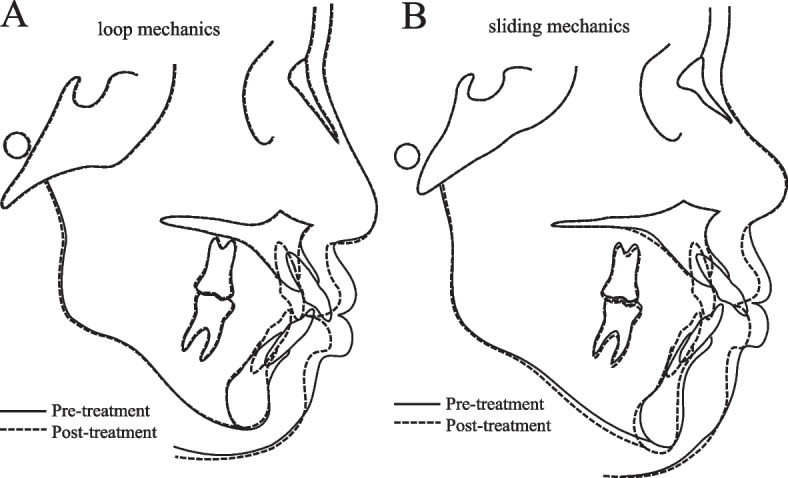
Typical treatment example with loop mechanics and sliding mechanics. Superimposition of pre-treatment and post-treatment of (**A**) loop mechanics, (**B**) sliding mechanics

Superimposition of lateral cephalograms in a typical case treated with loop mechanics is shown in Fig. 3A and with sliding mechanics is shown in Fig. 3B. Both anterior teeth were fully retracted, but in loop mechanics, the anterior teeth showed greater bodily movement and intrusion, and the lower anterior teeth were more upright and less intruded (Fig. 3A). In sliding mechanics, the anterior teeth were fully retracted, and the upper anterior teeth were intruded but more lingually tipped and FH to occlusal plane angle was opened, and lower anterior teeth were intruded (Fig. 3B).

## Discussion

A number of case reports of orthodontic treatment using a TAD have been published [5, 6, 17, 18], but there have been few studies examining actual treatment examples [19], the most of studies were based on in vitro finite element models of sliding mechanics and not of loop mechanics [13–16]. We usually use a 0.019 × 0.025-in archwire in loop mechanics and a 0.018 × 0.025-in archwire in sliding mechanics. In loop mechanics, a larger arch wire size is accessible compared to sliding mechanics, because of lower friction and diminished binding [12]. The larger size of the wire is more favorable to achieve lingual root torque during upper anterior tooth retraction, so, we frequently use loop mechanics in the patients especially need vertical and torque control of anterior teeth. Therefore, we examined the effects of traction form TADs on anterior tooth movement behavior by comparing between sliding and loop mechanics using treatment results from 20 actual patients with severe bimaxillary protrusion.

For the horizontal measurements taken in this study, there were significant differences between T0 and T1 in all denture measurements and Z-angle for both sliding and loop mechanics. In this study, variation of NA-U1 in loop group and sliding group was − 7.8 mm and − 9.2 mm, respectively. Variation of NB -L1 in loop group and sliding group was − 7.1 mm and − 5.9 mm respectively. These findings suggest that the extraction spaces were effectively used for anterior teeth retraction without mesial movements in the molar teeth. Moreover, these measurements surpassed the variation of anterior teeth retraction treated using conventional anchorage methods [20, 21]. These results strongly suggested that TADs are significantly effective methods for achieving substantial retraction of anterior teeth. It is known that TADs close with the root of the teeth, they tend to loosen and drop out [22]. Throughout the treatment period with both sliding and loop mechanics, there was no instance of TADs loosening or dropping out. This suggests little mesial movement of the molars.

However, a detailed comparison of the data indicated differences in tooth movement between the two groups.

There were significant differences in FMIA, NB to L1, and Z-angle between the two groups at T1. In loop mechanics, the lower anterior teeth were greatly retracted compared to the sliding group, which would result in a better profile change (Z-angle). These can be understood by comparing the variation of T1–T0 (Table 2). The upper incisors moved significantly more bodily movement in the loop group (FH to U1, − 15.0°; NA to U1, − 7.8 mm) compared with the sliding group (FH to U1, − 21.1°; NA to U1, − 9.2 mm), while the lower incisors moved significantly more upright in the loop group (FMIA, 17.5°; NB to L1, − 7.1 mm) compared with the sliding group (FMIA, 11.5°; NA to U1, − 5.8 mm). Furthermore, from the results of T1–T0 variation in vertical measurements (Table 3), the upper incisors were significantly more intruded (U1 edge to PP) in the loop group (− 1.3 mm), but they were extruded in the sliding group (0.7 mm), resulting in the maintenance of FH to the occlusal plane (0.3°) in the loop group but clockwise rotation of the occlusal plane (3.5°) in the sliding group. A comparison of typical treated examples for each type of treatment mechanics indicated differences in the behavior of the anterior teeth (Fig. 3A, B). The upper incisor was retracted and intruded with less lingual crown tipping and the occlusal plane was maintained in the loop group (Fig. 3A) compared to the sliding group (Fig. 3B). These results suggest that in the treatment of bimaxillary protrusion cases, by using loop mechanics to pull the maxillary incisors from near the center of resistance to the TAD, the upper and lower incisors moved to better positions in the horizontal and vertical directions compared to using sliding mechanics.

Tominaga et al. [13] reported that lingual crown tipping of the maxillary incisor was observed when the retraction force was applied at a height of 0 mm from the bracket slot during sliding treatment, and the direction of tipping changed from lingual crown tipping to lingual root tipping as the position of the retraction force on the power arm was moved apically from the blackest slot, and bodily movement occurred at a height of 11.6 mm from the 0.022-in blackest slot in finite element models. This result indicates that it is important to level the height of the TAD with the center of resistance of the anterior teeth during retraction.

In the present study of 20 cases undergoing retraction of anterior teeth, the TADs were placed at almost the same level with the center of resistance of the upper anterior teeth in both groups. However, in the sliding group, the upper anterior teeth exhibited more lingual crown tipping possibly due to the oblique traction vector from the hooks to the TADs. In contrast, in the loop group, the parallel retraction vectors were applied with closing loops at the same level as the TADs. Therefore, lingual crown tipping of the upper anterior teeth was minimized compared with the sliding group. These results partially support those of in vitro finite element models. It is considered that the traction from higher position using a long arm fixed between the lateral incisor and canine in sliding method will shows similar result as that of loop method. However, the larger size of arch wire was used for anterior teeth retraction in loop mechanics, which is more effective to prevent lingual crown tipping of upper anterior teeth. In addition, the closing loop of the upper arch wire with excessive Spee was opened by 1.0~1.5 mm by pulling the loop top from the TADs to tip the closing loop behind with a ligature wire for activation (Fig. 1A). Distal tipping of the closing loop with a ligature wire emphasized intrusion and lingual root torque forces in the anterior teeth, and these forces acted effectively on intrusion and lingual root tipping of the anterior teeth in the loop group. To summarize the results of study, the maintenance of the occlusal plane by loop mechanics may be due to the 1) larger arch wire size, 2) traction direction of higher position from the TADs, and 3) effective intrusion and lingual root torque force by tipping the closing loop.

One the other hand, the lower anterior tooth movement was different from the upper anterior teeth. The treatment mechanics during the first few months until re-leveling of the full arch in both groups were the same, but there were differences in the subsequent retraction mechanics of the anterior teeth. However, the lingual upright amount of lower anterior teeth was smaller and the intrusion amount of anterior teeth was larger in sliding mechanics compared to loop mechanics. Although the reason is not yet clear due to the lack of sequential lateral cephalograms, it is likely that some amount of intrusion of the lower anterior teeth occurred within the first few months, while the curve of Spee would be slightly flattened in both groups. In sliding group, lower anterior teeth may be further intruded, while the curve of Spee was more flattened by the flat sliding arch wire. On the other hand, in loop group, there may be little further intrusion of lower anterior teeth due to the buffering effect of the closing loop. They may explain the differences in behavior of lower anterior teeth according to the different mechanics, but additional studies are needed to explain these differences.

With regard to measurements of the skeletal pattern (e.g., SNA, SAB, ANB, and FMA), as there were no significant differences in any of the values between pre- and post-treatment, the reasons mentioned above and retraction mechanics used in this study appeared not to influence the skeletal pattern.

## Conclusions

In conclusion, both sliding and loop mechanics can be effectively applied to retract upper anterior teeth from TADs implanted in the posterior buccal alveolar bone.

However, the modality has several differences. An oblique retraction force vector with a lower point of application causes less intrusion and more lingual tipping of upper anterior teeth as well as more clockwise rotation of the occlusal plane. Conversely, a parallel retraction force vector with a point of application of the same level as a TAD leads to more intrusion and less lingual tipping of upper anterior teeth as well as better maintenance of the occlusal plane. It is also likely that intrusion and lingual root tipping of the upper anterior teeth were effectively performed by pulling the closing loop from the TAD for activation. Loop mechanics using larger wire size is useful for the treatment of patients who need vertical and torque control of anterior retraction.

Therefore, it is better to select a traction method according to the needs of each individual case.

## Data Availability

All data generated or analyzed during this study are included in this article.

## Abbreviation

TADs: Temporary anchorage devices
